# High-risk HPV and bacterial STIs in a primary screening population in rural Hainan, China: prevalence, co-infection, and association with cervical abnormalities

**DOI:** 10.3389/fpubh.2026.1773020

**Published:** 2026-02-23

**Authors:** Guoxuan Li, Zhe Lu, Feifei Yin, Hui Lu

**Affiliations:** 1 Department of Clinical Laboratory, Center for Laboratory Medicine, Affiliated Women and Children’s Medical Center of Hainan Medical University, Haikou, Hainan, China; 2 Hainan Medical University-The University of Hong Kong Joint Laboratory of Tropical Infectious Diseases, Key Laboratory of Tropical Translational Medicine of Ministry of Education, School of Basic Medicine and Life Sciences, Hainan Medical University, Haikou, Hainan, China; 3 Reproductive Medical Center, Hainan Women and Children’s Medical Center, Haikou, Hainan, China

**Keywords:** cervical intraepithelial neoplasia, *Chlamydia trachomatis*, high-risk human papillomavirus, public health strategies, *Ureaplasma urealyticum*

## Abstract

**Introduction:**

With the expansion of cervical cancer screening in China, clarifying the epidemiology of high-risk human papillomavirus (HR-HPV) and co-infecting reproductive tract bacterial pathogens in underserved regions is increasingly important. We investigated the prevalence and co-infection patterns of HR-HPV and bacterial pathogens (*Chlamydia trachomatis*, *Ureaplasma urealyticum*, *Neisseria gonorrhoeae*) among rural women in Hainan, and examined their associations with cervical epithelial lesions to inform optimized risk stratification for this population.

**Methods:**

Within a screening cohort of 8,925 women in Hainan (May–October 2025), we employed a nested case–control design enrolling 869 HR-HPV-positive cases and 473 negative controls, which were randomly selected from HR-HPV-negative women, with age adjustment applied in statistical analyses. Participants underwent genotyping for HR-HPV and bacterial sexually transmitted infections (*C. trachomatis*, *U. urealyticum*, *N. gonorrhoeae*). Cervical abnormalities were assessed via ThinPrep Cytologic Test and subsequent histopathology.

**Results:**

HR-HPV infection was associated with older age and lower educational attainment (*p* < 0.001). Among HR-HPV subtypes, single-type HR-HPV infection predominated (83.08%). *U. urealyticum* was the most prevalent reproductive tract bacterial pathogen (45.75%) and was more frequently co-detected in HR-HPV-positive women than in controls (*p* < 0.001). Network analysis further identified *U. urealyticum* as a central hub, showing strong co-occurrence with HR-HPV52 and HR-HPV58. Clinically, *C. trachomatis* was independently associated with higher odds of atypical squamous cells of undetermined significance (aOR = 2.82, *p* = 0.025), whereas *U. urealyticum* was associated with an increased risk of cervical intraepithelial neoplasia grade 1 (aOR = 1.79, *p* = 0.040).

**Conclusion:**

This study highlights a distinct co-infection ecosystem centered on *U. urealyticum* in rural women, where *C. trachomatis* and *U. urealyticum* drive differentiated risks for early cervical lesions. Consequently, public health strategies should prioritize this underrepresented rural population. Integrating targeted bacterial STIs testing into HPV screening could significantly enhance risk stratification and intervention efficiency in settings with limited resources.

## Introduction

Cervical cancer is one of the most common female malignancies in the world, ranking at the top among all female malignancies (1). Its incidence and mortality are still at a high level, and especially in developing countries, it poses a prominent threat to women’s health (2, 3). Persistent high-risk human papillomavirus (HR-HPV) infection is a major cause of cervical cancer initiation and progression, detectable in most cases (4–6). However, HR-HPV is not the only driver for the initiation and development of cervical cancer (7–10). Bacterial sexually transmitted pathogens in the reproductive tract may serve as vital co-risk factors, further leading to the initiation and development of cervical intraepithelial neoplasia and even cervical cancer through co-infection and interaction with HR-HPV (7–10). Of note, our previous research on patients in the gynecological outpatient clinic found that *Chlamydia trachomatis* and *Ureaplasma urealyticum* were detected more frequently in HPV-positive cervical samples, suggesting a certain tendency for co-infection with HPV (11).

HR-HPV, *C. trachomatis*, *U. urealyticum*, and *Neisseria gonorrhoeae* are pathogens of sexually transmitted infections (12–15). One of the important characteristics of sexually transmitted infections is its significant concealment, which can colonize at a low level for a long time or persist in the host without typical symptoms, thereby causing delayed diagnosis and treatment as well as increased risk of sustained transmission and difficulty of clinical identification without being easily detected (8, 16–18). Occult infections not only have adverse effects on individual reproductive health but also exacerbate the disease burden and complexity of prevention and control at the population level, which emerges as an urgent issue of concern in the field of global public health (8, 15, 19, 20). Previous studies on STIs have mainly focused on the outpatient population of Grade-A Tertiary Hospitals in cities (15, 21, 22). Our preliminary survey on 2,389 symptomatic women in Haikou City showed that the age group of 15–25 years is at high risk of infection, and mixed infections are correlated with an increased risk of HPV (11). However, such data based on passive hospital visits inevitably suffers from selection bias, making it difficult to represent the asymptomatic population in rural areas who may carry pathogens for a long time, thus potentially underestimating the true infection burden and co-infection patterns of this “occult infection” population. Especially in developing countries and rural underserved communities, STIs are often not adequately diagnosed and effectively managed due to limited access to healthcare services, socio-economic disparities, and cultural barriers, with the disease burden mainly borne by women in these areas (23–26). This urban–rural gap may be further driven by disparities in healthcare infrastructure and accessibility, as well as differences in screening coverage and testing strategies implemented in public health centers and primary healthcare facilities (27, 28).

Existing evidence suggests a possible synergistic promoting effect of *C. trachomatis* and *U. urealyticum* on the progression of cervical lesions (8, 13, 29). The inflammatory microenvironment induced by these pathogens may weaken local immune surveillance, providing favorable conditions for sustained HR-HPV infection and the progression of related lesions (8, 30–32). However, the specific roles exerted by *C. trachomatis* and *U. urealyticum* during this procedure are still controversial, and there is no consensus on whether they have direct carcinogenic potential (32–34). In light of this, this study aims to provide more direct pathological evidence to elucidate the correlations of *C. trachomatis* and *U. urealyticum* infection with the severity of cervical intraepithelial neoplasia.

From a geographical perspective, Hainan Island is the only tropical island province in China, which is geographically relatively independent, with certain natural environment and socioecological differences and isolation effects as compared to inland areas, and its pathogen epidemic characteristics may have regional specificity distinct from inland areas (35–37). Hainan is also a highly connected hub for tourism and population mobility, with frequent inflow and outflow through ports and transport corridors that may facilitate pathogen introduction and transmission. Such sustained mixing driven by travel and commerce may further amplify cross regional introduction and onward transmission of sexually transmitted pathogens. Complementing this mobility-driven pressure, the unique tropical ecological environment and high biodiversity of Hainan Island may provide a more unique ecological background for the transmission, colonization, and sustained prevalence of multiple pathogens (38–40). Studies in this context can help supplement existing evidence from a regional and ecological perspective and provide a scientific basis for the development and implementation of more targeted cervical health management strategies that integrate multiple pathogen screening in this region.

This study focuses on the asymptomatic female screening population in rural areas of Hainan Island, aiming to answer two key questions: firstly, to systematically characterize the prevalence and co-infection patterns of major STIs, and assess the impact of sociodemographic factors, such as age and educational attainment, as well as geographic heterogeneity, on the distribution of infection; secondly, to evaluate the association between specific STIs co-infection and the risk of significant cytological abnormalities and cervical precancerous lesions. Altogether, this study fills the evidence gap at the urban–rural and regional levels and improves the epidemiological landscape of related reproductive tract infections.

## Materials and methods

### Study design and participants

This study adopted a population-based case–control design, relying on the cervical cancer screening cohort in Hainan Province established between May and October 2025 (*N* = 8,925). All identified HR-HPV-positive individuals (*n* = 869) were enrolled in the case group to ensure high representativeness. For the control group, we randomly selected a subset of HR-HPV-negative women (*n* = 473), resulting in an approximate 1.8:1 case-to-control ratio. Regarding sample size adequacy, we conducted a post-hoc power analysis using the observed effect sizes as *a priori* parameters. The theoretical minimum sample sizes required for 80% power (*α* = 0.05) were calculated to be 420 for *C. trachomatis* (Cohen’s *h* = 0.310) and 186 for *U. urealyticum* (Cohen’s *h* = 0.413). Our actual sample size (*n* = 1,342) far exceeded these thresholds. Moreover, the post-hoc statistical power for these primary outcomes was confirmed to be >0.999, demonstrating robust statistical reliability despite the unbalanced design. For *N. gonorrhoeae*, given its low overall prevalence which resulted in expected frequencies of <5 in certain contingency table cells, Fisher’s exact test was employed to avoid bias associated with asymptotic statistical methods and to ensure the accuracy of inferences. During cervical cancer screening, all participants underwent HR-HPV genotyping and DNA testing for *C. trachomatis*, *U. urealyticum*, and *N. gonorrhoeae*. Additionally, ThinPrep Cytologic Test (TCT) was performed, followed by histopathological examination for individuals with abnormal colposcopy findings.

Inclusion criteria: (1) females who aged 35–64 years, consistent with the target population defined by the Implementation Plan for the “Two Cancers” Screening Project for Women in Hainan Province and the National Health Commission’s cervical cancer screening guidelines (41); (2) individuals with a history of sexual activity and who did not receive total hysterectomy; (3) individuals without vaginal irrigation, medication or operation within 24 h before screening as well as without sexual activity within 48 h; (4) individuals who can understand the research content, voluntarily participate in the study, and sign an informed consent form; (5) individuals without systemic or local antiviral/antibiotic treatment within the past 2 weeks. Exclusion criteria: (1) females who were pregnant or within 3 months postpartum; (2) individuals who underwent total hysterectomy in the past or were diagnosed with cervical cancer and received curative treatment; (3) individuals with obvious acute inflammatory lesions of the vagina or cervix during examination, clinical symptoms of unexplained abnormal vaginal bleeding, or those in the menstrual period; (4) individuals with severe heart, liver, kidney dysfunction or other severe systemic disorders; (5) individuals with mental or cognitive impairment who are unable to cooperate with various examinations; (6) a low-risk HPV infection indicated by the HPV genotyping test; (7) general demographic data or key clinical data are severely missing, making it impossible to conduct reliable statistical analysis.

### Sample collection

The screening of all participants conformed to the standardized cervical cancer screening protocol in Hainan Province, and exfoliated cervical cells were harvested (41). The cervix was exposed with a sterile vaginal speculum inserted by the clinical doctor. The swab was inserted into the posterior vaginal fornix and rotated clockwise 10 times to collect exfoliated cervical epithelial cells. Subsequently, the swab head carrying exfoliated cervical cells was placed into a cell tube (Hybribio Biochemistry Co., Ltd., China), sealed, and immediately preserved at 4 °C. Within 24 h, the samples were sent to the molecular diagnostic laboratory and pathology laboratory for HR-HPV genotyping, *C. trachomatis*, *U. urealyticum*, and *N. gonorrhoeae* DNA assays and TCT.

For pathological tissue sampling, the most typical lesion area was identified by clinical doctors under a colposcope, combined with acetic acid and iodine tests. Biopsy forceps were used for fixed-point or multi-point sampling (approximately 2–3 mm tissue blocks). Endocervical curettage was performed if necessary to screen for cervical canal lesions. The tissue was fixed in sufficient 10% neutral formalin within a few seconds to prevent autolysis and maintain cell morphology. All samples were accurately labeled with the sampling location and sent to the pathology laboratory for further processing. The transportation and storage of samples were carried out in accordance with uniform standards. The research subjects were fully informed of the research purposes and signed informed consent forms, which were reviewed and approved by the Ethics Committee of Hainan Women and Children’s Medical Center [Ethics Approval Number: HNWCMC (2023) Lunshen No. (76)].

### Research methods

#### Screening strategy

According to the 14 HR-HPV genotypes confirmed by the World Health Organization based on the cervical cancer screening documents in Hainan Province, this study categorized HPV16, 18, 31, 33, 35, 39, 45, 51, 52, 56, 58, 59, 66, and 68 as high-risk types (41); meanwhile, the LR-HPV samples detected here were excluded.

#### HR-HPV detection

The collected exfoliated cervical cells were subjected to HR-HPV genotyping. HPV infection and its genotypes were detected using a HPV nucleic acid typing test kit, which can simultaneously detect 23 HPV genotypes, including HPV16, 18, 31, 33, 35, 39, 45, 51, 52, 56, 58, 59, 66, 68, 6, 11, 42, 43, 44, 53, 81, 73, and 82.

#### *Chlamydia trachomatis*, *Ureaplasma urealyticum*, and *Neisseria gonorrhoeae* assays

The collected exfoliated cervical cells were tested for three pathogens, *C. trachomatis*, *U. urealyticum*, and *N. gonorrhoeae*, using the Daan Gene nucleic acid detection kits (batch No.: DA0070, DA0080, DA0060; Daan Gene Co., Ltd., Guangzhou, China). The operation procedures were strictly in accordance with the product manual requirements.

### Diagnostic performance

#### Cervical liquid-based cytology evaluation

TCT detection was carried out, and the results were interpreted and graded based on the Bethesda System (TBS). Cytological results were classified per TBS standards into: negative for intraepithelial lesion or malignancy, atypical squamous cells of undetermined significance (ASC-US), low-grade squamous intraepithelial lesion (LSIL), atypical squamous cells-cannot exclude high-grade squamous intraepithelial lesion (ASC-H), high-grade squamous intraepithelial lesion (HSIL), and cervical squamous cell carcinoma.

#### Histopathological diagnosis

According to the cervical cancer screening strategies in Hainan Province, for those with abnormalities detected by colposcopic screening, colposcopy-guided targeted biopsy should be performed on suspected lesion areas (41); if no clear lesions are found, biopsies (a four-point scale) are conducted at 3, 6, 9, and 12 o’clock in the cervical transformation zone, and if necessary, endocervical curettage is applied. All tissue samples were fixed in 10% neutral buffered formalin, routinely dehydrated, and paraffin-embedded, sliced into 4-μm-thick paraffin sections, and dyed with hematoxylin–eosin.

The images were reviewed independently by two pathologists with senior professional titles who were blinded to the HPV and TCT results of the subjects (double-blind) for a pathological diagnosis; an inconsistent diagnosis should be reviewed and confirmed by a third senior pathologist. Pathological diagnosis was classified according to the World Health Organization’s histological classification criteria of cervical tumors, including normal, LSIL (cervical intraepithelial neoplasia 1), HSIL (cervical intraepithelial neoplasia grade 2 and higher), and cervical squamous cell carcinoma.

### Statistical methods

All data analysis was completed with the application of RStudio software (R version 4.5.1) using packages including tidyverse (v2.0.0), dplyr (v1.1.4), and readxl (v1.4.5) for data management; logistf (v1.26.1), car (v3.1–3), and pROC (v1.19.0.1) for statistical modeling; and ggplot2 (v4.0.0) and ComplexHeatmap (v2.24.1) for data visualization. Microsoft Excel 2021, and statistical results were validated using IBM SPSS Statistics 27.0. Categorical variables were expressed in frequency and percentage [n (%)], and inter-group comparisons were realized using the Welch’s *t* test, exact binomial test, chi-square test, and Fisher’s exact test dependent on applicable conditions. Given the observed significant difference in age distribution between the HR-HPV-positive and negative groups, age was identified as a key confounding factor. Consequently, the inter-group comparison of pathogen prevalence was performed using analyses adjusted for age to account for this disparity. Subsequently, the associations between relevant risk factors and infection or cervical lesion outcomes were evaluated using unadjusted logistic regression models and models adjusted for age. To further validate the robustness of these associations, fully adjusted models were additionally fitted by incorporating age, education level, sampling site, HPV vaccination history, and HPV genotype risk groups (HR-HPV16/18, 52/58, and others). The data were expressed as an odds ratio (OR) and its 95% confidence interval (CI). Consistent with the primary regression analysis, receiver operating characteristic (ROC) curves were generated from predicted probabilities derived from both unadjusted models and models adjusted for age to evaluate predictive performance. The area under the ROC curve (AUC) was calculated using a nonparametric approach (pROC package in R), and AUC 95% CIs were estimated using DeLong’s method. Optimal cut-off values were determined by maximizing Youden’s index, with corresponding sensitivity and specificity reported. Differences in AUCs between single-variable and combined models were compared using DeLong’s test for correlated ROC curves (two-sided). The area under the ROC curve (AUC) was calculated using a nonparametric approach (pROC package in R), and AUC 95% CIs were estimated using DeLong’s method. Optimal cut-off values were determined by maximizing Youden’s index, with corresponding sensitivity and specificity reported. Differences in AUCs between single-variable and combined models were compared using DeLong’s test for correlated ROC curves (two-sided). A value of *p* < 0.05 (two-sided) was interpreted to denote statistical significance. The R scripts and processed datasets supporting all analyses in this study are available in a public GitHub repository to ensure reproducibility (42).

## Results

### Baseline characteristics and epidemiological landscape of HR-HPV

The average age of the HR-HPV-positive population was markedly higher than that of the HR-HPV-negative population (49.01 ± 8.68 years vs. 46.45 ± 8.23 years, *p* < 0.001). Given this significant disparity, age was included as a covariate for adjustment in all subsequent comparative and regression analyses. From the perspective of education level, significant differences were observed between the two groups (*p* < 0.001). The proportion of individuals with less than a high school education was significantly higher in the HR-HPV-positive group than in the negative group (87.6% vs. 77.2%). Conversely, the HR-HPV-negative group had a higher proportion of individuals with a high school education or above (22.8% vs. 12.4%), specifically driven by a higher prevalence of university-level education (14.8% vs. 4.9%). Significant regional distribution differences were observed between the two groups across sampling sites (*p* < 0.001), with a higher proportion of individuals from Chengmai County in the HR-HPV-positive group (41.8% *vs*. 10.6%) and a higher proportion of individuals from Ding’an County in the HR-HPV-negative group (46.7% *vs*. 32.2%); however, insignificant difference was noted in ethnic distribution between the two groups (*p* = 0.576); A significant difference was observed between the groups, with a greater proportion of vaccinated individuals in the HR-HPV-negative group (6.8% *vs*. 3.8%, *p* = 0.016) (see Table 1).

**Table 1:** Baseline characteristics.

Characteristics	HR-HPV-negative (*n* = 473)	HR-HPV-positive (*n* = 869)	*p* value
Age, years (Mean ± SD)	46.45 ± 8.23	49.01 ± 8.68	<0.001
35–39 years	120 (25.4)	171 (19.7)	0.016
40–44 years	100 (21.1)	138 (15.9)	0.016
45–49 years	76 (16.1)	132 (15.2)	0.671
50–54 years	77 (16.3)	133 (15.3)	0.639
55–59 years	64 (13.5)	172 (19.8)	0.004
60–64 years	36 (7.6)	123 (14.2)	<0.001
Ethnic minorities			0.576
Li	39 (8.2)	61 (7.0)	
Miao	4 (0.8)	14 (1.6)	
Zhuang	3 (0.6)	8 (0.9)	
Yao	–	1 (0.1)	
Mongol	–	1 (0.1)	
Educational level (%)			<0.001
University	70 (14.8)	43 (4.9)	
High school	38 (8.0)	65 (7.5)	
Less than high school	365 (77.2)	761 (87.6)	
Sampling site (%)			<0.001
Chengmai County	50 (10.6)	363 (41.8)	
Ding’an County	221 (46.7)	280 (32.2)	
Qiongzhong Li and Miao Autonomous County	74 (15.6)	116 (13.3)	
Wanning City	128 (27.1)	98 (11.3)	
Tunchang County	–	12 (1.4)	
Vaccination history (%)			0.016
Vaccinated	32 (6.8)	33 (3.8)	
Unvaccinated	441 (93.2)	836 (96.2)	

A total of 1,342 participants were included, with 473 HR-HPV–negative women and 869 HR-HPV–positive women. HR-HPV positivity was defined as positivity for any high-risk HPV genotype. Age is presented as mean ± standard deviation and was compared using Welch’s two-sample t test. Categorical variables are presented as counts and percentages. Group comparisons used Pearson’s chi-square test for age strata, education level, and vaccination history. Fisher’s exact test was applied when sparse cells were present for ethnicity and sampling site, and Monte Carlo simulation was used for sampling site. All tests were two-sided, and *p* < 0.05 was considered statistically significant. A dash indicates no observations.

### Distribution of HPV genotype multiplicity

**Figure 1:**
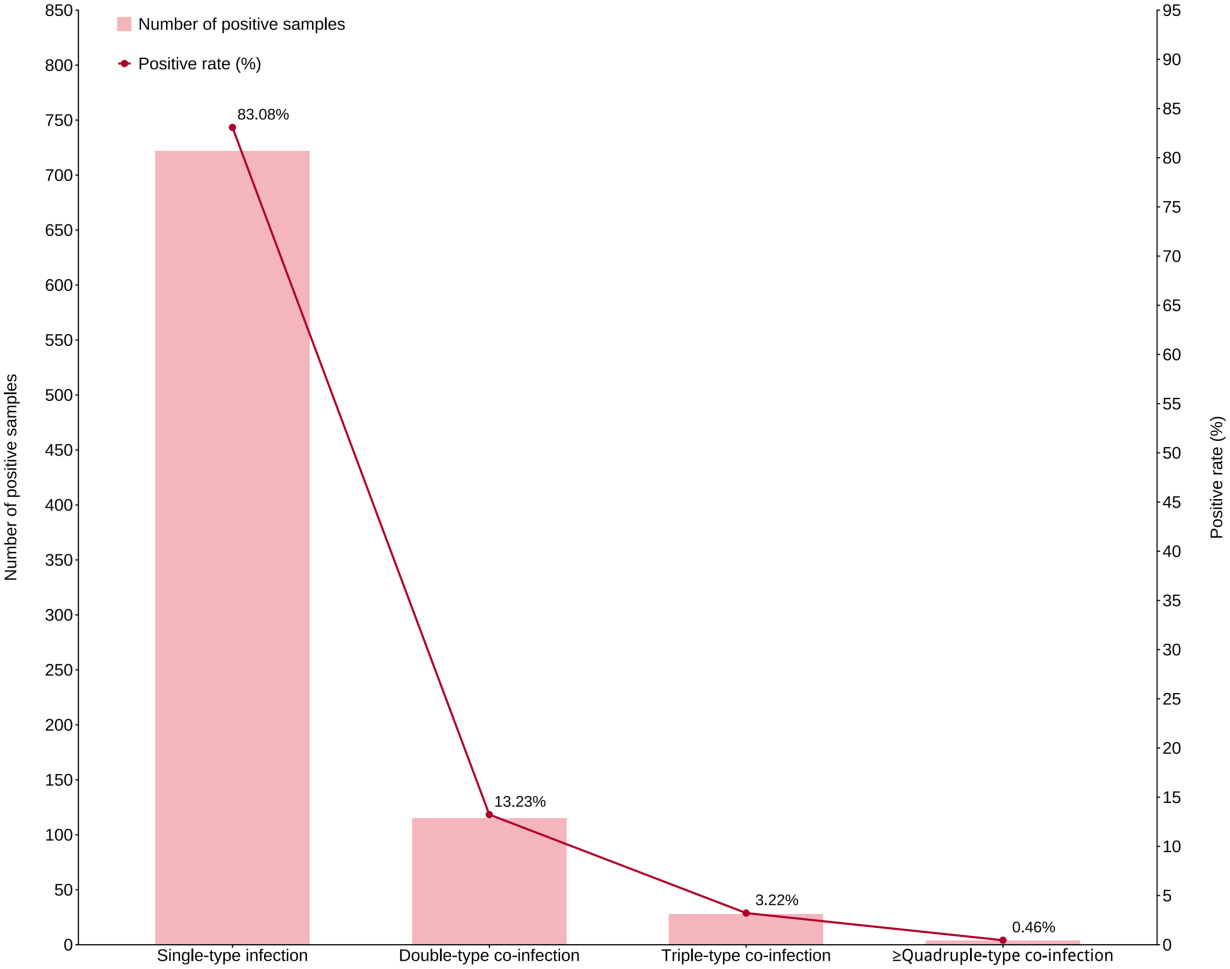
Distribution of HR-HPV genotype multiplicity strictly within the HR-HPV-positive population. The analysis characterizes the complexity of viral infections among HR-HPV-positive participants (*n* = 869) by categorizing cases based on the number of concurrent HR-HPV genotypes detected. The bar chart (left *y*-axis) represents the number of cases (*n*), while the line chart (right *y*-axis) indicates the constituent ratio (%). Single HR-HPV genotype infection was predominant (*n* = 722, 83.08%). Multiple HR-HPV genotype infections included dual infection (*n* = 115, 13.23%), triple infection (*n* = 28, 3.22%), and infection with ≥4 genotypes (*n* = 4, 0.46%). This highlights the pattern of viral genotype co-existence within the host.

Main attention was paid to the distribution characteristics of genotype multiplicity of viral sexually transmitted pathogens (HR-HPV). HR-HPV infection can be classified into single-genotype infection and mixed-genotype infection based on the genotyping results. The results summarized in Figure 1 showed that among 869 HR-HPV-positive cases, single infection with one HR-HPV subtype accounted for the highest proportion (722 cases, 83.08, 95% CI: 80.4–85.5%), followed by mixed infection with two HR-HPV subtypes (115 cases, 13.23, 95% CI: 11.1–15.7%), and mixed infection with three subtypes (28 cases, 3.22, 95% CI: 2.2–4.6%) or more subtypes (4 cases, 0.46, 95% CI: 0.1–1.2%).

### Epidemiological profile and age-specific prevalence of *C. trachomatis*, *U. urealyticum*, and *N. gonorrhoeae*

**Figure 2:**
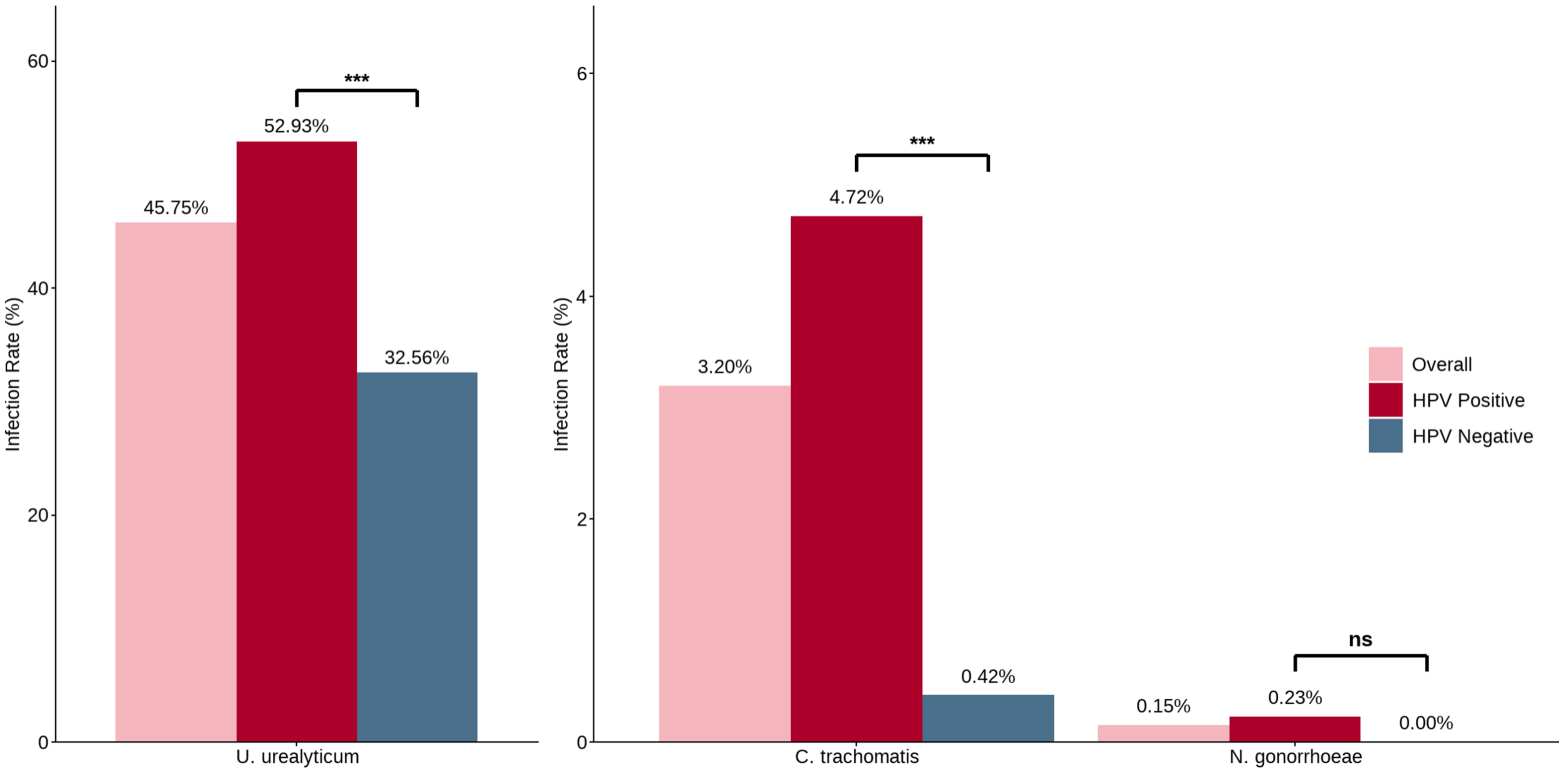
Prevalence of *C. trachomatis*, *U. urealyticum*, and *N. gonorrhoeae* in the total cohort and by HR-HPV status, with comparisons adjusted for age. The number of cases and infection rates for three bacterial STIs are shown for the total study population (*n* = 1,342), the HR-HPV-negative group (*n* = 473), and the HR-HPV-positive group (*n* = 869). *C. trachomatis*: overall prevalence was 3.20% (43/1,342); prevalence was significantly higher in the HR-HPV-positive group compared to the negative group (4.72% vs. 0.42%, *p* < 0.001). *U. urealyticum*: Overall prevalence was 45.75% (614/1,342); prevalence was significantly higher in the HR-HPV-positive group (52.93% vs. 32.56%, *p* < 0.001). *N. gonorrhoeae*: overall prevalence was 0.15% (2/1,342), with no statistically significant difference between groups (*p* > 0.05). Comparisons were performed using the *χ*^2^ test for *C. trachomatis* and *U. urealyticum*, and Fisher’s exact test for *N. gonorrhoeae*. *p* < 0.001; ns, not significant.

Simultaneous multi-pathogen screening showed that *U. urealyticum* was the dominant epidemic pathogen in this population, with a significantly higher infection rate than *C. trachomatis* and *N. gonorrhoeae* (45.75% *vs*. 3.20% *vs*. 0.15%, *p* < 0.001). Furthermore, analyses adjusted for age showed significantly higher infection rates of *C. trachomatis* and *U. urealyticum* in HR-HPV-positive individuals than in HR-HPV-negative individuals (*p* < 0.001), whereas no between-group difference was observed for *N. gonorrhoeae* (*p* > 0.05) (Figure 2).

**Figure 3:**
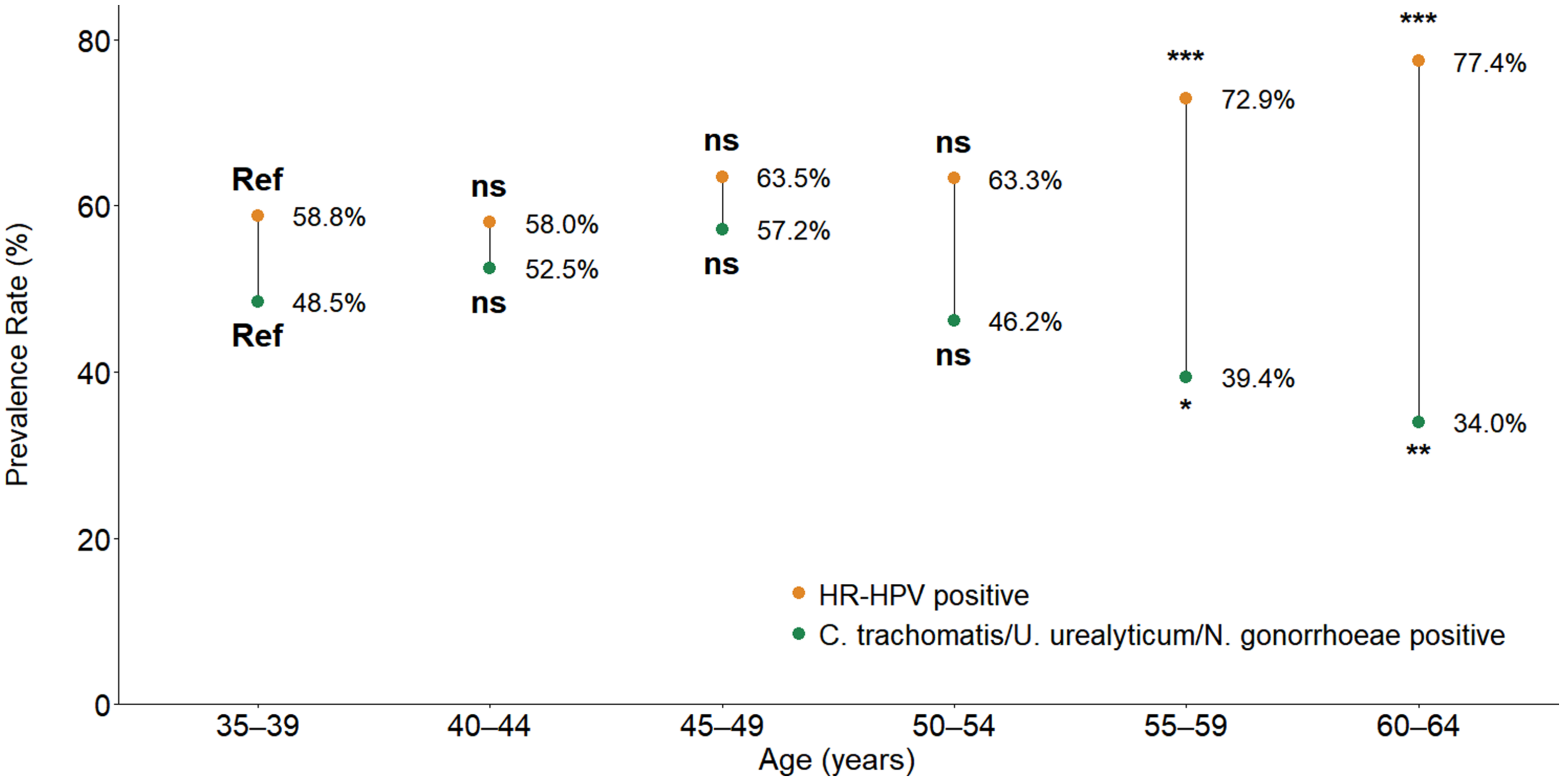
Age-specific prevalence trends of HR-HPV and genital bacterial STIs (*C. trachomatis/U. urealyticum/N. gonorrhoeae*). The study population (*n* = 1,342) was stratified into six age groups (from 35–39 to 60–64 years). HR-HPV prevalence (orange dots) showed an age-cumulative pattern, increasing significantly with age and peaking in the 60–64 age group (77.4%). Compared to the 35–39 reference group, differences were statistically significant for the 55–59 (p < 0.001) and 60–64 (*p* < 0.001) age groups. Bacterial STIs prevalence (green dots, defined as positive for *C. trachomatis, U. urealyticum, or N. gonorrhoeae*) showed a declining trend with age, decreasing from 48.5% in the 35–39 group to 34.0% in the 60–64 group. Compared to the reference group, significant decreases were observed in the 55–59 (*p* = 0.038) and 60–64 (*p* = 0.003) groups. *p*-values were calculated using the χ^²^ test (two-sided).

Subsequently, this research explored the dynamic distribution patterns of pathogens in this ecological niche by characterizing the infection age patterns of HR-HPV and *C. trachomatis*/*U. urealyticum/N. gonorrhoeae*. The two infections exhibited completely opposite trends: the HR-HPV infection rate was significantly increased with age, showing an “age-based accumulating” pattern (from 58.8% in the 35–39 age group to 77.4% in the 60–64 age group); whereas, the infection rate of *C. trachomatis*/*U. urealyticum/N. gonorrhoeae* showed a “age-based decreasing” pattern (from 48.5 to 34.0%) (Figure 3).

### Co-occurrence network analysis of HPV genotypes and *C. trachomatis*, *U. urealyticum*, and *N. gonorrhoeae*

**Figure 4:**
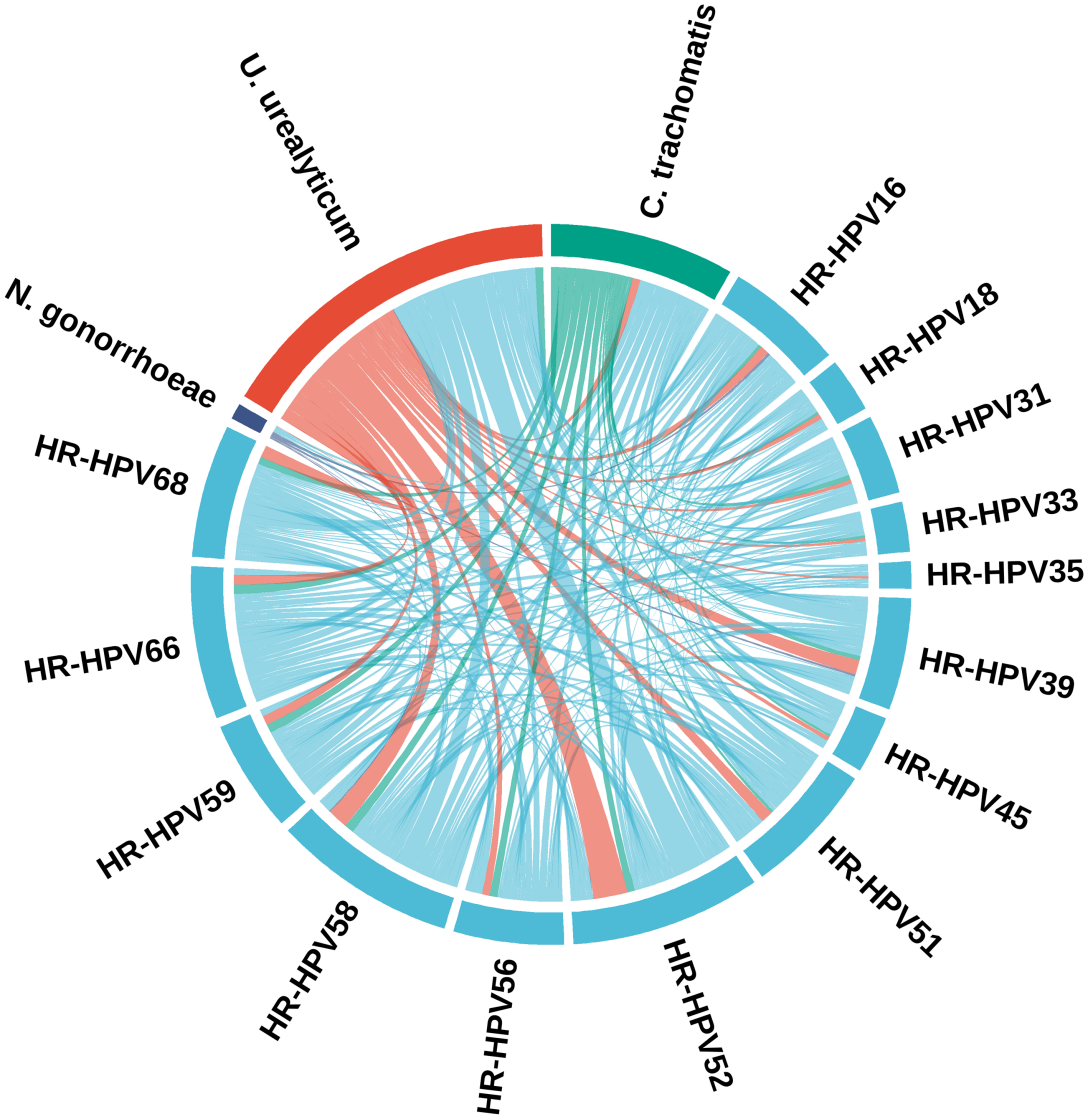
Chord diagram visualizing the pairwise co-occurrence network between HR-HPV genotypes and genital bacterial pathogens. Shows a chord diagram of co-occurrence patterns between HR-HPV genotypes and bacterial STIs in the HR-HPV-positive population. After normalization for pathogen prevalence, the outer arc length reflects the relative detection frequency of each pathogen, and the ribbon width reflects the relative extent of co-detection between pathogen pairs. *U. urealyticum* exhibits the largest sector and the widest ribbons, indicating the strongest network connectivity and a higher co-occurrence tendency with HR-HPV compared with other bacteria. In contrast, *C. trachomatis* and *N. gonorrhoeae* display fewer and weaker connections. The most prominent links are observed between *U. urealyticum* and HR-HPV52 and HR-HPV58.

Among the bacterial STIs, *U. urealyticum* displayed the strongest network connectivity characteristics. After normalizing for prevalence, it retained the largest peripheral sector and the widest connecting bands extending inward. This topology suggests that *U. urealyticum* has a significantly higher co-occurrence probability with HR-HPV strains compared to other bacteria. Conversely, the connections for *C. trachomatis* and *N. gonorrhoeae* were relatively limited and weaker, appearing as thinner ribbons in the network. Based on these topological features, *U. urealyticum* fits the characteristics of a “hub” node, serving as a major co-factor in the HR-HPV-positive population. Specifically, the strongest band widths were identified between *U. urealyticum* and genotypes HR-HPV52 and HR-HPV58, indicating a potential synergistic relationship between these specific pathogens (Figure 4).

**Figure 5:**
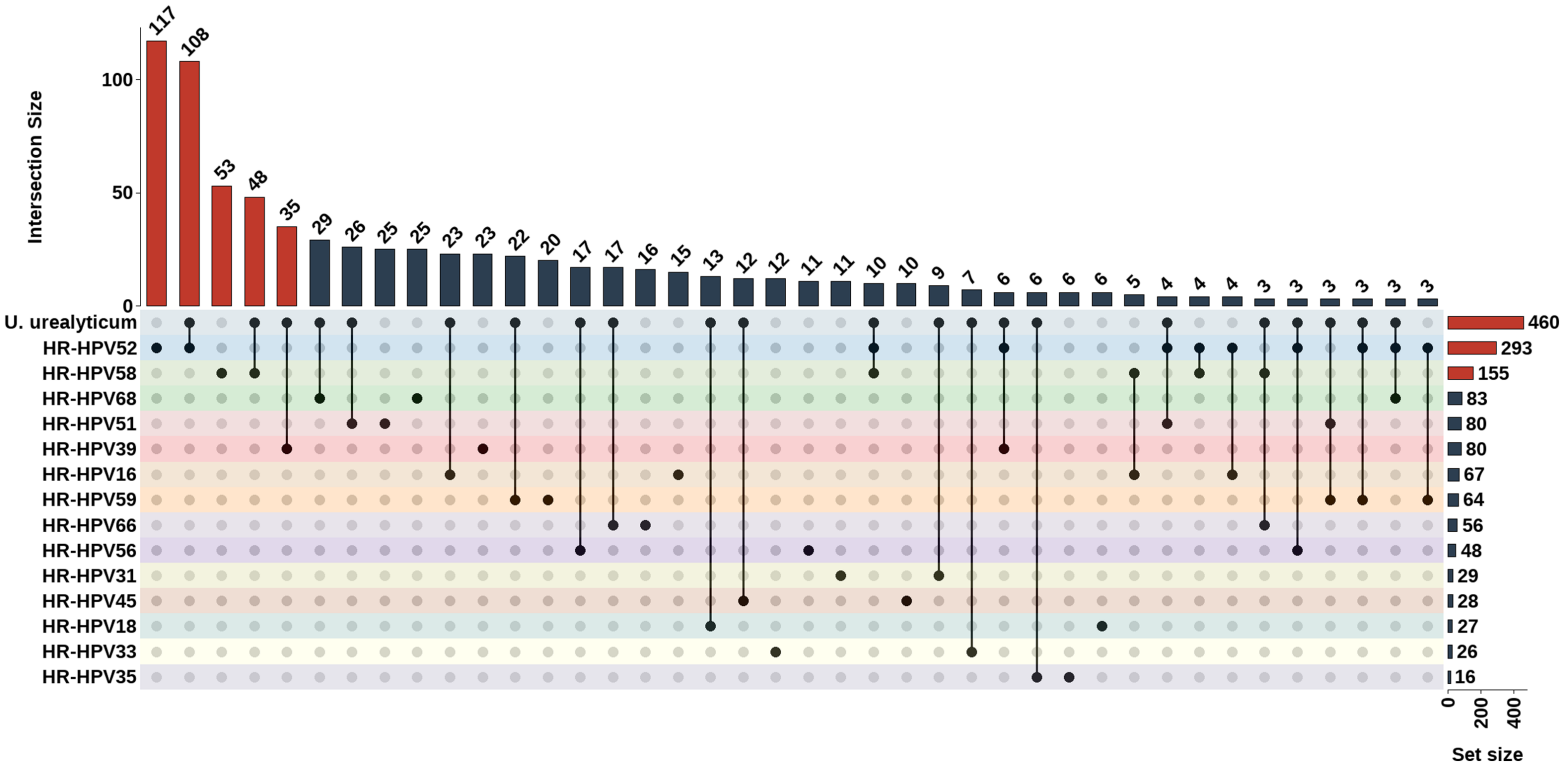
UpSet plot characterizing co-infection patterns between *U. urealyticum* and specific HR-HPV genotypes. The plot quantifies the intersection of *U. urealyticum* with various HR-HPV subtypes. The matrix at the bottom identifies the pathogen combinations (single or co-infections), with connected black dots representing the specific sets being intersected. The top vertical bar chart displays the number of samples in each intersection, while the right horizontal bar chart shows the total prevalence of each pathogen. The analysis highlights that HPV52 + *U. urealyticum* and HPV58 + *U. urealyticum* are the most dominant co-infection profiles among dual infections.

Among the HR-HPV-positive population (*n* = 869), HPV52 (*n* = 293, 33.72%) and HPV58 (*n* = 155, 17.84%) were high-risk types with higher detection rates. *U. urealyticum* positive individuals accounted for approximately half (52.93%) of the HR-HPV-positive population. In the co-infection cases, HR-HPV52 plus *U. urealyticum* (*n* = 108, 12.43%) and HR-HPV58 plus *U. urealyticum* (*n* = 48, 5.52%) constituted the most common co-infection patterns (Figure 5).

### Clinical association of *C. trachomatis* and *U. urealyticum* infections with cervical lesions

**Figure 6:**
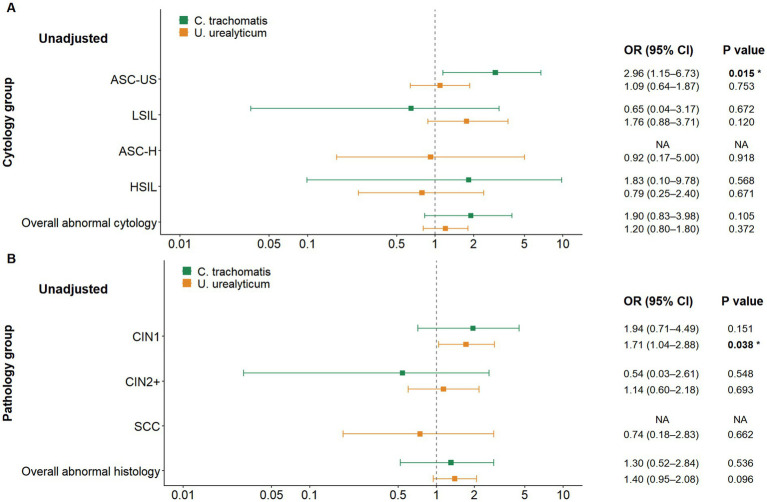
Forest plot of unadjusted logistic regression results among HR-HPV-positive women. **(A)** Associations of *C. trachomatis* and *U. urealyticum* with overall abnormal cytology and individual cytology categories. *C. trachomatis* is associated with increased odds of ASC-US, whereas neither pathogen is significantly associated with overall abnormal cytology when all abnormal categories are combined. **(B)** Associations of *C. trachomatis* and *U. urealyticum* with histopathological outcomes. *U. urealyticum* is associated with increased odds of CIN1, while no significant associations are observed for CIN2+ or overall abnormal histology. The vertical dashed line indicates an odds ratio of 1. NA indicates estimates could not be calculated due to sparse data.

**Figure 7:**
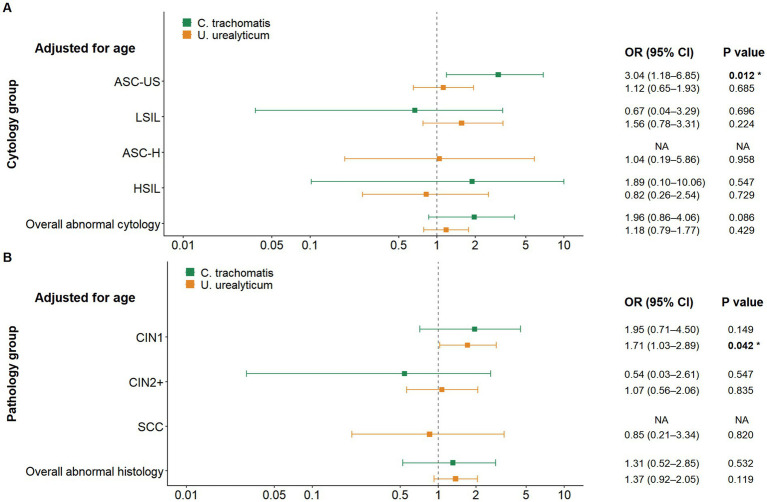
Forest plot of multivariable logistic regression results among HR-HPV-positive women, adjusted for age. **(A)** Associations of *C. trachomatis* and *U. urealyticum* with overall abnormal cytology and individual cytology categories. *C. trachomatis* is associated with increased odds of ASC-US, whereas neither pathogen is significantly associated with overall abnormal cytology when all abnormal categories are combined. **(B)** Associations of *C. trachomatis* and *U. urealyticum* with histopathological outcomes. *U. urealyticum* remains associated with increased odds of CIN1 in models adjusted for age, while no significant associations are observed for CIN2+ or overall abnormal histology. The vertical dashed line indicates an odds ratio of 1. NA indicates estimates could not be calculated due to sparse data.

To thoroughly assess the clinical associations and account for potential confounding variables, particularly age, in the HR-HPV-positive population, we conducted both univariate (unadjusted) and multivariable logistic regression analyses adjusted for age. In the context of cytological screening, when aggregating all categories of cytological abnormalities (ASC-US, LSIL, ASC-H, HSIL), neither *C. trachomatis* nor *U. urealyticum* infection was significantly associated with the overall risk of abnormalities in either the unadjusted models or the models adjusted for age. Nevertheless, a more detailed stratified analysis uncovered a specific association. *C. trachomatis* infection was associated with an increased risk of ASC-US in both the unadjusted analysis (OR = 2.96, *p* = 0.015) and the analysis adjusted for age (OR adjusted for age = 3.04, 95% CI 1.18–6.85, *p* = 0.012), whereas no significant associations were observed between *C. trachomatis* and other cytological abnormality categories. At the histopathological level, *U. urealyticum* infection was significantly associated with an increased risk of CIN1, and this association remained significant in both the unadjusted analysis (OR = 1.71, 95% CI 1.04–2.88, *p* = 0.038) and the analysis adjusted for age (OR adjusted for age = 1.71, 95% CI 1.03–2.89, *p* = 0.042) (see Figures 6, 7; Table 2).

**Table 2:** Univariate analysis.

Clinical group	Outcome (vs reference)	Pathogen	Crude OR	95% CI	*p* value
Cytology group					
ASC-US	ASC-US vs. NILM	*C. trachomatis*	2.96	1.15–6.73	0.015
		*U. urealyticum*	1.09	0.64–1.87	0.753
LSIL	LSIL vs. NILM	*C. trachomatis*	0.65	0.04–3.17	0.672
		*U. urealyticum*	1.76	0.88–3.71	0.120
ASC-H	ASC-H vs. NILM	*C. trachomatis*	NA	NA	NA
		*U. urealyticum*	0.92	0.17–5.00	0.918
HSIL	HSIL vs. NILM	*C. trachomatis*	1.83	0.10–9.78	0.568
		*U. urealyticum*	0.79	0.25–2.40	0.671
Overall abnormal cytology	(ASC-US/LSIL/ASC-H/HSIL) vs. NILM	*C. trachomatis*	1.90	0.83–3.98	0.105
		*U. urealyticum*	1.20	0.80–1.80	0.372
Pathology group					
CIN1	CIN1 vs. CIN0	*C. trachomatis*	1.94	0.71–4.49	0.151
		*U. urealyticum*	1.71	1.04–2.88	0.038
CIN2+	CIN2+ vs. CIN0	*C. trachomatis*	0.54	0.03–2.61	0.548
		*U. urealyticum*	1.14	0.60–2.18	0.693
SCC	SCC vs. CIN0	*C. trachomatis*	NA	NA	NA
		*U. urealyticum*	0.74	0.18–2.83	0.662
Overall abnormal histology	(CIN1/CIN2+/SCC) vs. CIN0	*C. trachomatis*	1.30	0.52–2.84	0.536
		*U. urealyticum*	1.40	0.95–2.08	0.096

Univariate associations of *C. trachomatis* and *U. urealyticum* infections with cervical lesions. Crude OR, crude odds ratio; CI, confidence interval. ASC-US, atypical squamous cells of undetermined significance; LSIL, low-grade squamous intraepithelial lesion; ASC-H, atypical squamous cells cannot exclude HSIL; HSIL, high-grade squamous intraepithelial lesion; NILM, negative for intraepithelial lesion or malignancy; CIN, cervical intraepithelial neoplasia; SCC, squamous cell carcinoma. “NA” indicates values not estimable due to small cell counts (sparse data) in the model.

To further validate the robustness of these findings, we expanded the multivariable model (Table 3) to strictly control for a comprehensive set of confounders. In addition to age, we explicitly adjusted for education level and sampling site to serve as proxies for socioeconomic status and healthcare access (43, 44), HPV vaccination history to account for immunological protection (45), and HPV genotype risk groups (stratified into types 16/18, 52/58, and other high-risk types) to control for varying viral pathogenicity (45–47). Notably, the results from this fully adjusted model were highly consistent with the estimates adjusted for age. The association between *C. trachomatis* and ASC-US remained significant (aOR = 2.82, 95% CI 1.06–6.66, *p* = 0.025), as did the risk of CIN1 associated with *U. urealyticum* (aOR = 1.79, 95% CI 1.04–3.16, *p* = 0.040).

**Table 3:** Multivariable analysis.

Clinical group	Outcome (vs reference)	Pathogen	Adjusted OR	95% CI	*p* value
Cytology group					
ASC-US	ASC-US vs. NILM	*C. trachomatis*	2.82	1.06–6.66	0.025
		*U. urealyticum*	1.05	0.61–1.85	0.851
LSIL	LSIL vs. NILM	*C. trachomatis*	0.82	0.04–4.28	0.851
		*U. urealyticum*	1.62	0.77–3.54	0.213
ASC-H	ASC-H vs. NILM	*C. trachomatis*	NA	NA	NA
		*U. urealyticum*	1.05	0.19–5.95	0.955
HSIL	HSIL vs. NILM	*C. trachomatis*	1.26	0.07–6.77	0.830
		*U. urealyticum*	0.86	0.33–2.20	0.745
Overall abnormal cytology	(ASC-US/LSIL/ASC-H/HSIL) vs. NILM	*C. trachomatis*	1.91	0.82–4.10	0.111
		*U. urealyticum*	1.15	0.76–1.75	0.505
Pathology group					
CIN1	CIN1 vs. CIN0	*C. trachomatis*	2.27	0.80–5.59	0.094
		*U. urealyticum*	1.79	1.04–3.16	0.040
CIN2+	CIN2+ vs. CIN0	*C. trachomatis*	0.64	0.04–3.37	0.674
		*U. urealyticum*	1.07	0.53–2.19	0.848
SCC	SCC vs. CIN0	*C. trachomatis*	NA	NA	NA
		*U. urealyticum*	0.93	0.15–5.35	0.930
Overall abnormal histology	(CIN1/CIN2+/SCC) vs. CIN0	*C. trachomatis*	1.59	0.59–3.72	0.319
		*U. urealyticum*	1.37	0.88–2.14	0.168

Multivariable associations of *C. trachomatis* and *U. urealyticum* infections with cervical lesions. Models were adjusted for age, education, sampling site, vaccination history, and HPV genotype. Adjusted OR, adjusted odds ratio; CI, confidence interval. ASC-US, atypical squamous cells of undetermined significance; LSIL, low-grade squamous intraepithelial lesion; ASC-H, atypical squamous cells cannot exclude HSIL; HSIL, high-grade squamous intraepithelial lesion; NILM, negative for intraepithelial lesion or malignancy; CIN, cervical intraepithelial neoplasia; SCC, squamous cell carcinoma. “NA” indicates values not estimable due to small cell counts (sparse data) in the model.

### Predictive performance of *C. trachomatis* and *U. urealyticum* for early cervical lesions

**Figure 8:**
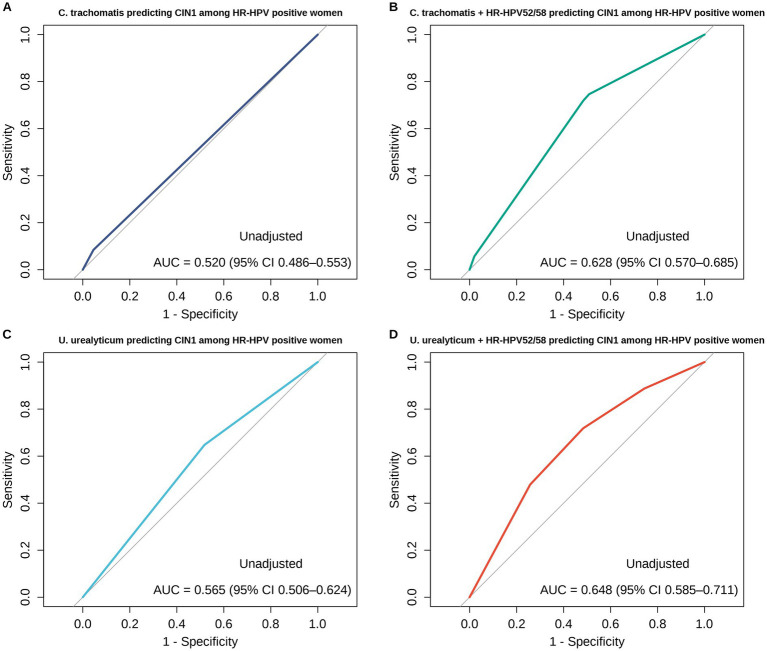
Receiver operating characteristic curves, unadjusted, for predicting CIN1 versus CIN0 among HR-HPV-positive women with histopathology-confirmed CIN0 or CIN1. **(A)** *C. trachomatis* alone, AUC 0.520 with 95% CI 0.486–0.553. **(B)** *C. trachomatis* plus HR-HPV52 and HR-HPV58, AUC 0.628 with 95% CI 0.570–0.685, with an AUC increase by paired DeLong test, *Z* = −3.603, *p* < 0.001. **(C)** *U. urealyticum* alone, AUC 0.565 with 95% CI 0.506–0.624. **(D)** *U. urealyticum* plus HR-HPV52 and HR-HPV58, AUC 0.648 with 95% CI 0.585–0.711, with an AUC increase by paired DeLong test, *Z* = −2.682, *p* = 0.007. The optimal probability cut-off was determined using Youden’s index.

**Figure 9:**
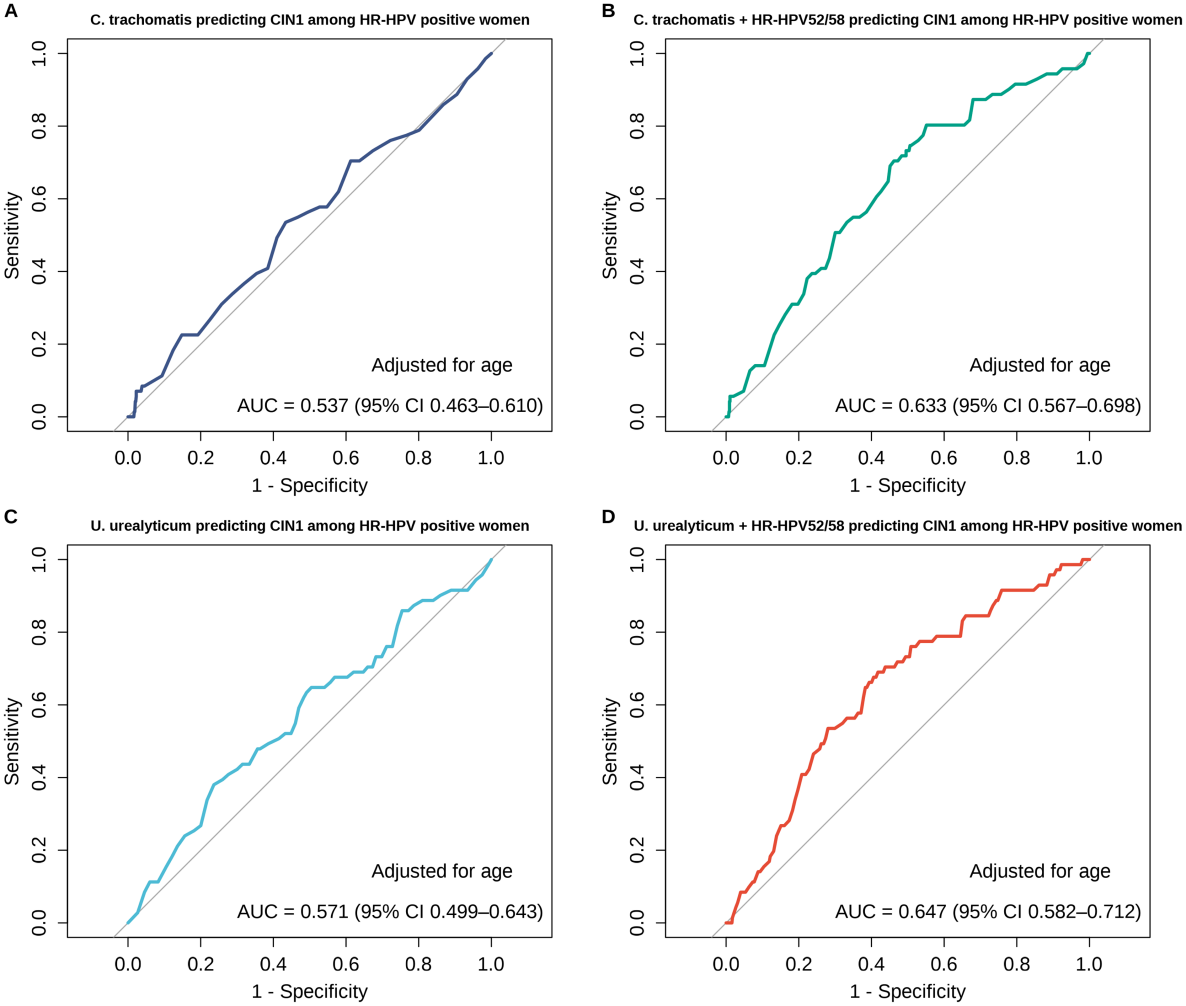
Receiver operating characteristic curves, adjusted for age, for predicting CIN1 versus CIN0 among HR-HPV-positive women with histopathology-confirmed CIN0 or CIN1: **(A)** *C. trachomatis* adjusted for age, AUC 0.537 with 95% CI 0.463–0.610; **(B)** *C. trachomatis* plus HR-HPV52 and HR-HPV58 adjusted for age, AUC 0.633 with 95% CI 0.567–0.698, with an AUC increase by paired DeLong test, *Z* = −2.867, *p* = 0.004; **(C)** *U. urealyticum* adjusted for age, AUC 0.571 with 95% CI 0.499–0.643; **(D)** *U. urealyticum* plus HR-HPV52 and HR-HPV58 adjusted for age, AUC 0.647 with 95% CI 0.582–0.712, with a borderline AUC increase by paired DeLong test, *Z* = −1.814, *p* = 0.070. The optimal probability cut-off was determined using Youden’s index.

Building upon the high prevalence and topological synergy of HPV 52/58 and *U. urealyticum* identified in the co-infection network, ROC analyses were conducted to evaluate the discrimination of CIN1 versus CIN0 among HR-HPV-positive women with histopathology-confirmed CIN0/CIN1, comparing models based on *U. urealyticum* or *C. trachomatis* alone and their combinations with HPV52/58 (Figure 8). In the unadjusted analyses, the AUC for *U. urealyticum* alone was 0.565 (95% CI 0.506–0.624) and increased to 0.648 (95% CI 0.585–0.711) after adding HPV52/58, with a significant AUC gain by paired DeLong test (*Z* = −2.682, *p* = 0.007). For *C. trachomatis*, the AUC increased from 0.520 (95% CI 0.486–0.553) for *C. trachomatis* alone to 0.628 (95% CI 0.570–0.685) after adding HPV52/58 (*Z* = −3.603, *p* < 0.001). Using Youden’s index to define the optimal probability cut-off, the threshold/sensitivity/specificity were 0.085/0.648/0.482 for *U. urealyticum* alone, 0.077/0.718/0.517 for *U. urealyticum* plus HPV52/58, 0.117/0.085/0.955 for *C. trachomatis* alone, and 0.069/0.746/0.491 for *C. trachomatis* plus HPV52/58. To assess robustness to confounding, we further fitted logistic regression models adjusted for age (Figure 9). In analyses adjusted for age, the AUC for *U. urealyticum* was 0.571 (95% CI 0.499–0.643) and increased to 0.647 (95% CI 0.582–0.712) after adding HPV52/58, with a borderline improvement (*Z* = −1.814, *p* = 0.070). For *C. trachomatis*, the AUC increased from 0.537 (95% CI 0.463–0.610) to 0.633 (95% CI 0.567–0.698) after adding HPV52/58, indicating a significant improvement (*Z* = −2.867, *p* = 0.004). The Youden-derived threshold/sensitivity/specificity were 0.106/0.380/0.764 for *U. urealyticum* (adjusted for age), 0.093/0.690/0.582 for *U. urealyticum* plus HPV52/58 (adjusted for age), 0.085/0.535/0.566 for *C. trachomatis* (adjusted for age), and 0.049/0.803/0.449 for *C. trachomatis* plus HPV52/58 (adjusted for age).

## Discussion

The retrospective analysis of rural women in Hainan Island provided insights into the epidemiological landscape of HR-HPV and other common bacterial sexually transmitted pathogens of the reproductive tract in a tropical island region. Among the 1,342 female participants, HR-HPV-positive individuals were older and had lower levels of education, with significant differences in infection rates across different cities and counties. Among the bacterial sexually transmitted pathogens detected in the reproductive tract, *U. urealyticum* showed the highest detection rate, followed by *C. trachomatis*. Meanwhile, in viral sexually transmitted pathogens, the majority of HR-HPV-positive individuals were infected with a single genotype; in the co-infection patterns of sexually transmitted pathogens, the co-infection of *U. urealyticum* and HR-HPV52/58 was the most common. It should be noted that in this study, *C. trachomatis* was linked to ASC-US, while *U. urealyticum* infection correlated with an enhanced risk of CIN1. These results suggest that co-infection of HR-HPV with *C. trachomatis* and *U. urealyticum* may synergistically affect the local inflammatory response and immune microenvironment, thereby correlating with the risk of occurrence and progression of early cervical lesions.

This retrospective analysis revealed that lower levels of education and residence in rural areas were related to higher detection rates of co-infection with HR-HPV and *C. trachomatis*, *U. urealyticum*, and *N. gonorrhoeae*. This finding is consistent with previous research, which suggests that highly educated individuals generally have better compliance with cervical cancer screening and are more inclined to adopt protective sexual activities (48–50). The results of this research suggested that females with limited educational opportunities may have a longer time interval between infection and diagnosis, allowing gradual accumulative co-infections. Geographical clustering also affects the distribution of infections, and economic inequality and inadequate access to medical services may contribute to the sustained transmission of HR-HPV and *C. trachomatis*, *U. urealyticum*, and *N. gonorrhoeae* in resource-limited areas.

Additionally, we observed a significantly higher average age of HR-HPV-positive females as compared to HR-HPV-negative females (49.01 years vs. 46.45 years), indicating that persistent HR-HPV infection may have an age-dependent cumulative increase. This finding is consistent with the “bimodal” age distribution of HPV infection reported in previous population studies: HPV infection exhibits a high incidence in young females and then decreases with age, with a second peak around 50–60 years; this phenomenon may be correlated with factors such as birth cohort effect, hormonal fluctuations during perimenopause, immunosenescence, and reactivation of latent HPV (51–54). On the contrary, we observed that the infections of *C. trachomatis*, *U. urealyticum*, and *N. gonorrhoeae* generally decreased with age.

The phenomenon mentioned above may be relevant to the current urban sexual health education and HPV vaccination strategies, mainly covering adolescents and people of early reproductive age; in contrast, a considerable proportion of females in rural areas were born before the “vaccine era,” and they are currently in the high-risk age window for cervical cancer and its precancerous lesions. The middle-aged and older adult population had limited access to standardized prevention services in the past, coupled with the hidden nature of infections and insufficient medical accessibility, which may lead to a long-term underestimation of their reproductive tract infection burden and related risks, thus becoming a weak link and “blind spot” of risk in the grassroots public health service system (55–57). Therefore, it is necessary to further tilt public health policy formulation and resource allocation towards middle-aged and older females in rural areas with scarce resources, making them the key target audience of cervical health management; in the meantime, the screening strategy should gradually shift from “opportunistic screening” triggered by medical visits to “full-coverage screening” based on population management, and improve the accessibility and efficiency of early diagnosis and treatment of precancerous lesions through standardized follow-up and stratified intervention, thereby reducing the long-term burden of cervical diseases in rural areas.

In this study cohort, the total detection rates of *C. trachomatis*, *U. urealyticum*, and *N. gonorrhoeae* were 3.20, 45.75, and 0.15%, respectively. The infection rates of *C. trachomatis* and *U. urealyticum* were remarkably higher in HR-HPV-positive samples than in HR-HPV-negative samples (*p* < 0.01), which was consistent with our previous research findings on gynecological outpatient patients (11). However, it is noteworthy that this study focuses on the asymptomatic female population, and the incidence of *U. urealyticum* remains strikingly high in this population. This phenomenon reminds us widespread occult transmission of *U. urealyticum* among “healthy” populations, and these asymptomatic carriers may have long been overlooked as potential sources of infection. The traditional detection and therapeutic strategies are only for symptomatic populations and may have limitations. We need to re-examine the prevention and control strategies against reproductive tract pathogens, strengthen public health education, and correct the misconception that “no symptoms mean no infection”. In contrast, the infection rate of *N. gonorrhoeae* was extremely low, basically consistent with the overall level shown by domestic surveillance data, but lower than 1–2% reported in some studies on the urban population (58, 59). This may also be related to the inclusion of a healthy female population in this study. Generally speaking, *N. gonorrhoeae* infection is often accompanied by more obvious clinical symptoms, and such patients are more likely to seek medical attention in medical institutions rather than “health screening”. Therefore, the infection rate of *N. gonorrhoeae* was relatively low in the healthy female population surveyed in this study.

In HR-HPV-positive individuals, *U. urealyticum* exhibited more prominent co-infection clustering characteristics compared to *C. trachomatis*, indicating a closer co-occurrence relationship with HR-HPV. Therefore, we incorporated the UpSet graph and co-occurrence chord diagram for conjoint analysis to characterize the ecological niche and topological characteristics of *U. urealyticum* in the HR-HPV co-infection network. The results displayed that HR-HPV52 and HR-HPV58 were dominant genotypes in rural populations of Hainan Island, forming the main co-infection pattern together with *U. urealyticum*, suggesting that these two may have specific biological synergistic effect, that is, *U. urealyticum* infection may provide “boosting” conditions for the invasion, colonization, or persistence of specific HR-HPV subtypes by altering the reproductive tract microenvironment, thereby performing a potential synergistic promoting role in the viral persistence and its pathogenic process. Therefore, *U. urealyticum*, as a key hub in the HR-HPV infection network, has important clinical guidance significance. In clinical practice, the detection of *U. urealyticum* should be regarded as not only a single pathogen infection but also as a “sentinel signal,” indicating that the patient may be in a complicated multi-pathogen exposure environment or a state of microecologic dysbiosis, thus requiring heightened alertness of clinical doctors and comprehensive intervention.

In this regard, we probed into the clinical relationship between *U. urealyticum* and cervical epithelial lesions. It has been reported that co-infection with *U. urealyticum* considerably raises the incidence of high-grade squamous intraepithelial neoplasia and invasive cervical cancer (8). A cross-sectional study in Brazil demonstrated that *Ureaplasma parvum*-positive females were significantly linked to the incidence of CIN1, and its co-infection with HR-HPV further augments the risk of CIN1 (8, 14). This study unveiled that *U. urealyticum* infection was associated with an increased risk of CIN1 (aOR = 1.79). *U. urealyticum* infection is often manifested with occult and chronic colonization characteristics, which rarely cause typical acute symptoms, making it more likely to continuously shape the local cervical microenvironment over a long period. Its long-term existence may interfere with epithelial cell homeostasis and repair processes through low-grade but sustained inflammatory stimulation and immune microenvironment remodeling, which further affects the fine regulation of cell cycle-related pathways, thereby providing a “soil” for the occurrence and maintenance of mild epithelial abnormalities. Therefore, from a pathological perspective, *U. urealyticum* appears to be more closely associated with low-grade lesions, typified by CIN1. In HR-HPV-positive individuals, this coinfection may be linked to the maintenance of pathological states characteristic of early-stage lesions. Consequently, we speculate that *U. urealyticum* might play a synergistic role in the progression of HR-HPV-associated cervical disease. Additionally, we did not observe a significant association between *U. urealyticum* infection and the risk of high-grade lesions or invasive cervical cancer. This result may be limited by both the research population and sample size. Firstly, this cohort mainly covered healthy individuals, who have a lower baseline probability of developing high-grade lesions than patients with clinical symptoms. Secondly, the number of high-grade lesions and cancer cases in the cohort was relatively small, resulting in insufficient statistical power and making it difficult to detect possible moderate or weak correlations.

Of note, although the overall positive rate of *C. trachomatis* is low (3.20%), it is more strongly linked to cytological abnormalities. Multivariable analysis suggested a significant correlation between *C. trachomatis* infection and ASC-US (aOR = 2.82). *C. trachomatis* infection often triggers relatively acute inflammatory responses, which can cause transient reactive changes and increased morphological heterogeneity in cervical epithelial cells. This type of inflammation-associated “reactive atypia” is more easily categorized into ASC-US in TCT detection and evaluation from the perspective of cytology. Furthermore, *C. trachomatis* infection may indirectly promote the susceptibility and persistent infection of HPV through the pathological “inflammation-barrier breakdown-immune microenvironment remodeling” axis, leading to a higher incidence of mild abnormalities (ASC-US) in cytology (31, 32, 60). Specifically, *C. trachomatis*-induced cervical inflammation weakens the integrity of the mucosal epithelial barrier and alters the local innate and adaptive immune status, creating favorable conditions for the colonization, replication, and sustained existence of HR-HPV (9, 31, 32, 34). While the discriminatory performance of *C. trachomatis* or *U. urealyticum* infection for cervical lesions is limited in the overall HR-HPV-positive population, co-testing significantly enhances predictive efficacy within the specific subgroup of HR-HPV 52/58 positive individuals. However, that given the overall low-to-moderate AUC values, these markers are best utilized as adjunctive tools within existing triage strategies. They aim to refine risk stratification rather than serving as independent decision-making criteria. Crucially, from the perspective of large-scale population screening, even this moderate statistical improvement translates into significant public health benefits. By refining risk stratification, this co-testing approach enables more precise resource allocation in settings with limited resources. For instance, regarding high-risk subgroups such as those co-infected with *U. urealyticum* and HPV52/58, clinical management can be optimized by intensifying surveillance through increasing the frequency of follow-up visits or shortening the intervals for colposcopy referrals. This strategy thereby maximizes the detection of early-stage lesions while avoiding over-management of low-risk populations.

Therefore, we recommend incorporating combined testing for *C. trachomatis* and *U. urealyticum* into the risk management workflow for HR-HPV 52/58 positive individuals, particularly those presenting with cytological abnormalities, persistent infection, or other high-risk factors. For patients with identified co-infections, intensified surveillance (e.g., shortened follow-up intervals) should be implemented, with prioritized referral for colposcopy where resources permit. Conversely, for those without co-infection and at lower overall risk, a relatively conservative management strategy may be adopted alongside standard follow-up. This stratified management approach aids in avoiding unnecessary overtreatment and alleviating patient psychological burden, thereby advancing cervical cancer screening toward precision and personalization.

Despite its significant public health implications, this study has certain limitations. Regarding the construction of the control group, due to cost-effectiveness considerations associated with multi-pathogen molecular screening in a large-scale population, we did not perform universal testing on the entire HR-HPV-negative cohort. Instead, we employed a random sampling strategy with an approximate 1.8:1 case-to-control ratio. However, *a priori* sample size estimation based on observed effect sizes confirmed that the actual sample size (*n* = 1,342) far exceeded the theoretical minimum (*n* = 420) required to satisfy statistical testing requirements. Further post-hoc power analyses revealed that the statistical power for the primary pathogens, *C. trachomatis* and *U. urealyticum*, approached 1.0 (>0.99). This suggests a negligible probability of Type II errors (false negatives) even with the current sample size, ensuring the high robustness of our conclusions. Although statistical power for *N. gonorrhoeae* was limited due to its low prevalence, exact probability tests (Fisher’s exact test) were applied to ensure the accuracy of statistical inferences. The control group was an unbiased subset selected from the same source cohort based on strict random sampling principles. Given the relative stability of baseline infection rates for genital pathogens in the general population, the current control sample size was sufficient to establish a reliable baseline reference. Furthermore, we acknowledge the observed age disparity between the case and control groups (49.01 ± 8.68 vs. 46.45 ± 8.23 years, *p* < 0.001). While this variance was rigorously controlled for as a covariate to eliminate confounding effects across all key analyses, including inter-group comparative assessments, multivariate logistic regression, and ROC predictive modeling, future investigations would benefit from implementing stricter age-matching designs during the recruitment phase to further minimize potential residual confounding. Beyond these design considerations, the specific molecular mechanisms by which *C. trachomatis* or *U. urealyticum*, together with HR-HPV, synergistically expedite the occurrence and progression of cervical cancer have not been fully elucidated. Longitudinal cohort studies and *in vitro*/*in vivo* functional experiments are necessary for further verification of their causal relationship and interpretation of potential mechanistic pathways. In addition, the limited number of high-level lesions and cancer cases restricts the analysis of associations with more severe diseases.

Another important consideration is that several potentially relevant determinants of co-infection were not captured in the current dataset and may have contributed to the observed heterogeneity in co-infection rates. Information on sexual risk-related behaviors, including the number of sexual partners and condom use, was not routinely collected during cervical cancer screening registration and therefore could not be incorporated into our analyses. Because these factors may influence population exposure patterns and co-infection transmission dynamics, future screening registration and follow-up systems should incorporate standardized behavioral data collection to better inform targeted public health interventions.

Moreover, our virological assessment primarily focused on HR-HPV, precluding evaluation of the potential contribution of LR-HPV to infection dynamics and lesion trajectories. Prior studies have suggested that, compared with HR-HPV infection alone, women with concomitant LR-HPV detection may have a lower subsequent risk of invasive cervical cancer, raising the possibility that LR-HPV co-infection could partially delay or attenuate HR-HPV-driven carcinogenic progression (61). At the molecular level, E2 proteins from both HR-HPV and LR-HPV types have been reported to broadly suppress innate antiviral pathways, including RIG-I/MDA5-MAVS, TLR3-TRIF, cGAS-STING, and JAK–STAT signaling, thereby facilitating viral persistence (62). However, existing evidence largely derives from studies of individual HPV types, and direct data demonstrating clear synergistic or antagonistic effects of HR-HPV/LR-HPV co-infection on immune evasion remain limited.

Taken together, future work integrating LR-HPV genotyping with prospective follow-up and mechanistic profiling of molecular and immune features is warranted to clarify whether LR-HPV co-detection modifies HR-HPV persistence and its associations with cervical epithelial lesions. At the population level, such studies could also compare HPV co-infection patterns between clinic-based populations and the general population to better characterize differences associated with healthcare-seeking contexts. Concurrently, metagenomic sequencing of cervicovaginal samples may further elucidate microbial community structure and functional pathways, providing deeper insights into the underlying mechanisms.

## Conclusion

Among rural female populations in Hainan Island, a tropical island region, STIs infection shows the characteristic of a public health issue intertwined with multiple factors, and its epidemic distribution is jointly shaped by demographic characteristics, behavioral factors, and regional differences. In this population, *U. urealyticum* is the main prevalent bacterial sexually transmitted pathogen and exhibits a directional co-infection pattern of *U. urealyticum* plus HR-HPV52/58. More importantly, *C. trachomatis* and *U. urealyticum* may participate in the occurrence and evolution of early cervical lesions through biological pathways, thus playing different roles in the natural history of the disease. Hence, in the future, the formulation of cervical disease prevention and control policies and resource allocation will further tilt towards healthy female populations and rural areas with relatively scarce resources, rendering them as key target audience of cervical health management. More importantly, it is necessary to go beyond the single perspective of “only focusing on HPV” and further integrate joint screening and stratified intervention for the key reproductive tract pathogens on the basis of optimizing HR-HPV screening strategies, so as to enhance the sensitivity of risk identification and improve early targeted management, achieving more efficient and sustainable cervical health protection.

## Data Availability

The original contributions presented in the study are included in the article/supplementary material, further inquiries can be directed to the corresponding authors.
