# Correction: High-risk HPV and bacterial STIs in a primary screening population in rural Hainan, China: prevalence, co-infection, and association with cervical abnormalities

**DOI:** 10.3389/fpubh.2026.1825486

**Published:** 2026-05-01

**Authors:** Guoxuan Li, Zhe Lu, Feifei Yin, Hui Lu

**Affiliations:** 1 Department of Clinical Laboratory, Center for Laboratory Medicine, Affiliated Women and Children's Medical Center of Hainan Medical University, Haikou, Hainan, China; 2 Hainan Medical University-The University of Hong Kong Joint Laboratory of Tropical Infectious Diseases, Key Laboratory of Tropical Translational Medicine of Ministry of Education, School of Basic Medicine and Life Sciences, Hainan Medical University, Haikou, Hainan, China; 3 Reproductive Medical Center, Hainan Women and Children's Medical Center, Haikou, Hainan, China

**Keywords:** cervical intraepithelial neoplasia, Chlamydia trachomatis, high-risk human papillomavirus, public health strategies, Ureaplasma urealyticum

The content of Tables 2 and 3 was incorrect in the published article. The data contents of Table 2 and Table 3 were swapped (i.e., Table 2 displayed the data of Table 3, and Table 3 displayed the data of Table 2). The correct tables appear below.

**Table 2:** Univariate analysis.

Clinical group	Outcome (vs. reference)	Pathogen	Crude OR	95% CI	*p* value
Cytology group					
ASC-US	ASC-US vs. NILM	*C. trachomatis*	2.96	1.15–6.73	0.015
		*U. urealyticum*	1.09	0.64–1.87	0.753
LSIL	LSIL vs. NILM	*C. trachomatis*	0.65	0.04–3.17	0.672
		*U. urealyticum*	1.76	0.88–3.71	0.120
ASC-H	ASC-H vs. NILM	*C. trachomatis*	NA	NA	NA
		*U. urealyticum*	0.92	0.17–5.00	0.918
HSIL	HSIL vs. NILM	*C. trachomatis*	1.83	0.10–9.78	0.568
		*U. urealyticum*	0.79	0.25–2.40	0.671
Overall abnormal cytology	(ASC-US/LSIL/ASC-H/HSIL) vs. NILM	*C. trachomatis*	1.90	0.83–3.98	0.105
		*U. urealyticum*	1.20	0.80–1.80	0.372
Pathology group					
CIN1	CIN1 vs. CIN0	*C. trachomatis*	1.94	0.71–4.49	0.151
		*U. urealyticum*	1.71	1.04–2.88	0.038
CIN2+	CIN2+ vs. CIN0	*C. trachomatis*	0.54	0.03–2.61	0.548
		*U. urealyticum*	1.14	0.60–2.18	0.693
SCC	SCC vs. CIN0	*C. trachomatis*	NA	NA	NA
		*U. urealyticum*	0.74	0.18–2.83	0.662
Overall abnormal histology	(CIN1/CIN2+/SCC) vs. CIN0	*C. trachomatis*	1.30	0.52–2.84	0.536
			*U. urealyticum*	1.40	0.95–2.08	0.096

Univariate associations of *C. trachomatis* and *U. urealyticum* infections with cervical lesions.

Crude OR, crude odds ratio; CI, confidence interval. ASC-US, atypical squamous cells of undetermined significance; LSIL, low-grade squamous intraepithelial lesion; ASC-H, atypical squamous cells cannot exclude HSIL; HSIL, high-grade squamous intraepithelial lesion; NILM, negative for intraepithelial lesion or malignancy; CIN, cervical intraepithelial neoplasia; SCC, squamous cell carcinoma. “NA” indicates values not estimable due to small cell counts (sparse data) in the model.

**Table 3:** Multivariable analysis.

Clinical group	Outcome (vs. reference)	Pathogen	Adjusted OR	95% CI	*p* value
Cytology group					
ASC-US	ASC-US vs. NILM	*C. trachomatis*	2.82	1.06-6.66	0.025
		*U. urealyticum*	1.05	0.61-1.85	0.851
LSIL	LSIL vs. NILM	*C. trachomatis*	0.82	0.04-4.28	0.851
		*U. urealyticum*	1.62	0.77-3.54	0.213
ASC-H	ASC-H vs. NILM	*C. trachomatis*	NA	NA	NA
		*U. urealyticum*	1.05	0.19-5.95	0.955
HSIL	HSIL vs. NILM	*C. trachomatis*	1.26	0.07-6.77	0.830
		*U. urealyticum*	0.86	0.33-2.20	0.745
Overall abnormal cytology	(ASC-US/LSIL/ASC-H/HSIL) vs. NILM	*C. trachomatis*	1.91	0.82-4.10	0.111
		*U. urealyticum*	1.15	0.76-1.75	0.505
Pathology group					
CIN1	CIN1 vs. CIN0	*C. trachomatis*	2.27	0.80-5.59	0.094
		*U. urealyticum*	1.79	1.04-3.16	0.040
CIN2+	CIN2+ vs. CIN0	*C. trachomatis*	0.64	0.04-3.37	0.674
		*U. urealyticum*	1.07	0.53-2.19	0.848
SCC	SCC vs. CIN0	*C. trachomatis*	NA	NA	NA
		*U. urealyticum*	0.93	0.15-5.35	0.930
Overall abnormal histology	(CIN1/CIN2+/SCC) vs. CIN0	*C. trachomatis*	1.59	0.59-3.72	0.319
			*U. urealyticum*	1.37	0.88-2.14	0.168

Multivariable associations of *C. trachomatis* and *U. urealyticum* infections with cervical lesions. Models were adjusted for age, education, sampling site, vaccination history, and HPV genotype. Adjusted OR, adjusted odds ratio; CI, confidence interval. ASC-US, atypical squamous cells of undetermined significance; LSIL, low-grade squamous intraepithelial lesion; ASC-H, atypical squamous cells cannot exclude HSIL; HSIL, high-grade squamous intraepithelial lesion; NILM, negative for intraepithelial lesion or malignancy; CIN, cervical intraepithelial neoplasia; SCC, squamous cell carcinoma. “NA” indicates values not estimable due to small cell counts (sparse data) in the model.

The original version of this article has been updated.

